# Personalized heterogeneous deformable model for fast volumetric registration

**DOI:** 10.1186/s12938-017-0321-3

**Published:** 2017-02-20

**Authors:** Weixin Si, Xiangyun Liao, Qiong Wang, Pheng Ann Heng

**Affiliations:** 1 Department of Computer Science and Engineering, The Chinese University of Hong Kong, Shatin, N.T. Hong Kong; 2 Shenzhen Key Laboratory of Virtual Reality and Human Interaction Technology, Shenzhen Institutes of Advanced Technology, Chinese Academy of Sciences, 1068 Xueyuan Avenue, 503644 Shenzhen, China

**Keywords:** Biomechanical deformable volumetric registration, Tissue–tissue coupling, Data-driven parameters estimation, Coarse-to-fine scheme

## Abstract

**Background:**

Biomechanical deformable volumetric registration can help improve safety of surgical interventions by ensuring the operations are extremely precise. However, this technique has been limited by the accuracy and the computational efficiency of patient-specific modeling.

**Methods:**

This study presents a tissue–tissue coupling strategy based on penalty method to model the heterogeneous behavior of deformable body, and estimate the personalized tissue–tissue coupling parameters in a data-driven way. Moreover, considering that the computational efficiency of biomechanical model is highly dependent on the mechanical resolution, a practical coarse-to-fine scheme is proposed to increase runtime efficiency. Particularly, a detail enrichment database is established in an offline fashion to represent the mapping relationship between the deformation results of high-resolution hexahedral mesh extracted from the raw medical data and a newly constructed low-resolution hexahedral mesh. At runtime, the mechanical behavior of human organ under interactions is simulated with this low-resolution hexahedral mesh, then the microstructures are synthesized in virtue of the detail enrichment database.

**Results:**

The proposed method is validated by volumetric registration in an abdominal phantom compression experiments. Our personalized heterogeneous deformable model can well describe the coupling effects between different tissues of the phantom. Compared with high-resolution heterogeneous deformable model, the low-resolution deformable model with our detail enrichment database can achieve 9.4× faster, and the average target registration error is 3.42 mm, which demonstrates that the proposed method shows better volumetric registration performance than state-of-the-art.

**Conclusions:**

Our framework can well balance the precision and efficiency, and has great potential to be adopted in the practical augmented reality image-guided robotic systems.

## Background

Surgical procedures are traditionally supported with pre-operative images, such as the computed tomography (CT) images and magnetic resonance (MR) images. The image quality can be very good, while when it comes to the surgical procedures, the link between images and patient is lost. In this regard, it can be intuitively displayed as an overlay of pre-operative images onto the patient’s body, which creates an augmented reality environment that enables surgeons to visualize the structures of interest [1]. Imaging looks inside the patient’s body, exposing the patient’s anatomy beyond what is visible on the surface.

A variety of methods have been developed to provide intra-operative image registration, which can be mainly classified into rigid and non-rigid registration. Rigid registration is generally applied when the target anatomy fulfills the criterion of rigidity and spatial distortions are not introduced in the image acquisition process [2]. It is a relatively quick and straightforward process that uses a rigid motion model with rotation and translation parameters of the target objects where tissue deformation can be ignored. Unfortunately, purely rigid transformation is not sufficient to describe the mechanical behaviors of human organ for most of the surgeries. To this end, this technique cannot produce an optimal alignment when human organ undergoes deformations caused by external forces (such as surgical tools) or natural motions (such as respiration). In such cases, non-rigid registration is required when the imaged anatomy non-rigidly deforms between acquisitions, which can provide a relatively accurate alignment for cases of non-rigid deformations. Readers can refer to  [3] for a thorough and comprehensive introduction of non-rigid registration.

Moreover, non-rigid registration can be broadly classified as either surface registration or volumetric registration. Both of these two approaches have advantages and weaknesses. Surface registration methods [4–10] have been shown to accurately align the highly complex morphological details on the surface of the human organ. Although these methods can offer the possibility to achieve visually coherent surface registration, they are limited to surface overlay without considering heterogeneous internal structures, such as vessels and tumors. In this regard, it is crucial to consider volumetric registration [11, 12]. Generally, volumetric registration methods can provide a correspondence field across the entire human organ, including common target regions (such as tumors and vessels) that are not in the domain of the surface-based alignment procedures.

Biomechanical model has proven to be a promising way for non-rigid volumetric registration [11, 13–20], which can accurately estimate the motion of in-depth volumetric structures. The finite element method (FEM) is the most widely used physics-based approach for developing deformable model, which can accurately describe the mechanical behaviors of human organ as continuous medium. Many researchers have reported certain success in achieving accurate volumetric registration based on FEM model. However, conventional FEM is so complicated that makes solution procedure time-consuming, which limits its application in clinical practice [21]. In this paper, we employ the total Lagrangian explicit dynamics (TLED) FEM to model the mechanical response of human organ under interactions. This is because TLED FEM is an efficient numerical algorithm that is based on the FEM while using the total Lagrangian formulation, where stresses and strains are measured with respect to the original configuration which allows for pre-computing of most spatial derivatives before the commencement of the time-stepping procedure [22]. Besides, TLED FEM is capable of handling both geometric and material non-linearities, which is beneficial to perform the large deformation analysis induced by tool–tissue interactions. In addition, the accuracy of the FEM relies heavily on the quality of geometric models meshes, while the geometric models of human organs are often complicated and irregular for representing the morphological details of the organs. Here we directly extract the uniform hexahedral mesh from the segmented medical images, which can greatly reduce the complexity of volumetric geometric mesh reconstruction and provide the high-quality mesh for biomechanical modeling.

In this paper, we are motivated to achieve fast volumetric registration for both surface and in-depth anatomical structures, where we have to determine the location of target region with great accuracy and avoid damaging the vessels which are needed for the post-operative rehabilitation of human organs. The contributions of this paper are as follows:

- *Personalized heterogeneous deformable model*. Our deformable model is based on a uniform and high-resolution hexahedral mesh directly extracted from the MR images, which is beneficial for the accuracy of TLED FEM analysis. A novel and effective tissue–tissue coupling strategy based on penalty method is proposed to model the in-depth anatomical structure of deformable body, and the personalized tissue–tissue coupling parameters are estimated in a data-driven way.
- *Coarse-to-fine scheme*. We propose a coarse-to-fine scheme to reduce the computational complexity of heterogeneous deformable model for fast volumetric registration. In more detail, we perform the TLED FEM on low-resolution hexahedral mesh first and then synthesize the microstructures using a detail enrichment database constructed by the high-resolution heterogeneous deformable model.

## Methods

### Materials

**Fig. 1:**
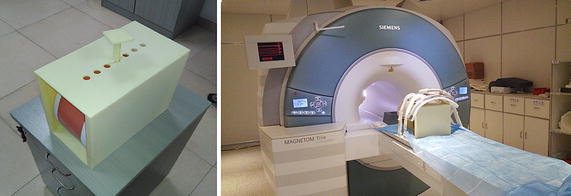
Experiment configurations

In this paper, we employ the triple modality 3D abdominal phantom (Model 057A, Computerized Imaging Reference Systems, Inc.) as the experimental object. It is a plastic model for medical education usage including artificial liver, vessels and tumors. In addition, the anatomical structures of the phantom can be identified by 3D MR images, which are acquired using Siemens 3T MAGNETOM Trio system in Paul C. Lauterbur Research Center For Biomedical Imaging, Shenzhen Institutes of Advanced Technology, Chinese Academy of Sciences. The scanned images of the phantom being compressed is used as the ground truth to validate the proposed volumetric registration method. The phantom has the advantage of providing controllable boundary conditions, and has been widely used as the experimental material to verify the registration method such as some other similar work of Lavely et al. [23] and Serban et al. [24]. To acquire 3D MR images of abdominal phantom under different compressions, we build a containing device by 3D printing technique to facilitate applying displacements during the MR imaging. The compression positions are on top of phantom and the MR scanning scene are shown in Fig. 1. We totally obtained 6 image datasets which consist of a dataset of initial status and 5 datasets of compressions (marked as *A*, *B*, *C*, *D*, *E*), and each dataset consists of 111 MR images. In addition, we label 25 landmarks (11 landmarks on liver surface and 14 internal landmarks inside the liver) in the initial status dataset and 5 compression datasets as groundtruth. Those landmarks are selected according to the structure of the tissues or organs, such as the internal surface or corners.

### Hexahedral mesh construction

In this paper, we focus on the cases when lesions (such as tumors) are present inside the parenchyma, these can be taken into account also from the mechanical point of view as they usually introduce significant heterogeneity. This heterogeneity can be straightforwardly included in the model through volumetric mesh. In this regard, hexahedral mesh or tetrahedral mesh  [11–13] are often employed rather than triangular mesh. Note that traditional volumetric registration methods are based on reconstructed tetrahedral mesh, while the reconstruction process may cause loss of precision. Considering that the segmented MR images are pixel level representation of the scanned phantom, we can avoid this limitation by directly constructing the hexahedral mesh. More importantly, we concentrate on improving the efficiency of volumetric registration, however, the tetrahedral mesh is usually irregular and contains distorted elements when deforms [25, 26], which requires re-meshing and results in huge computational cost and makes the simulation time-consuming [27, 28]. While the hexahedral mesh is known to be efficient in terms of stability and computational cost [25, 26], and hexahedral mesh presents better accuracy and efficiency than tetrahedral mesh in solid mechanics and structural engineering problems [29].

**Fig. 2:**
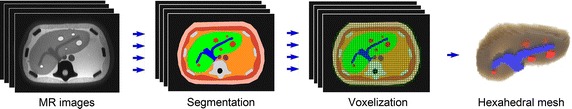
Hexahedral mesh construction

Thus, we directly construct a uniform high-resolution hexahedral mesh from the 3D MR images of vascularized liver. First, we manually segment the 3D MR images of vascularized liver, which can be classified as parenchyma, vessels and tumors. By setting the resolution ($74\times 18\times 54$), we divide each MR images into voxels and construct the uniform hexahedral mesh by connecting voxels in each MR image. The hexahedral mesh construction process can be observed in Fig. 2. Note that each hexahedron can only serve as one kind of tissue element, and the relationship between landmarks and the hexahedral mesh are established using the landmarks’ pixel coordinates.

It is worth noting that there are truly jaggy structures in our method around the hexahedral boundary no matter what kind of resolution it uses, and this will degrades the performance of our method in a certain degree. However, this is a kind of inevitable precision loss in the process of geometric mesh generation, even for the commonly adopted triangular mesh or tetrahedral mesh, since converting the raw medical images to surface/volumetric mesh leads to the loss of precision. Theoretically, the higher resolution the hexahedral mesh is, the more accurate registration method is. In this regard, we adopt a relative high resolution $74 \times 18 \times 54$ to reduce the precision loss induced by jaggy structures as much as possible. Meanwhile, the coarse-to-fine scheme proposed in this paper guarantees our registration efficiency is not influenced by the high-resolution hexahedral mesh.

### Personalized heterogeneous deformable model

**Fig. 3:**
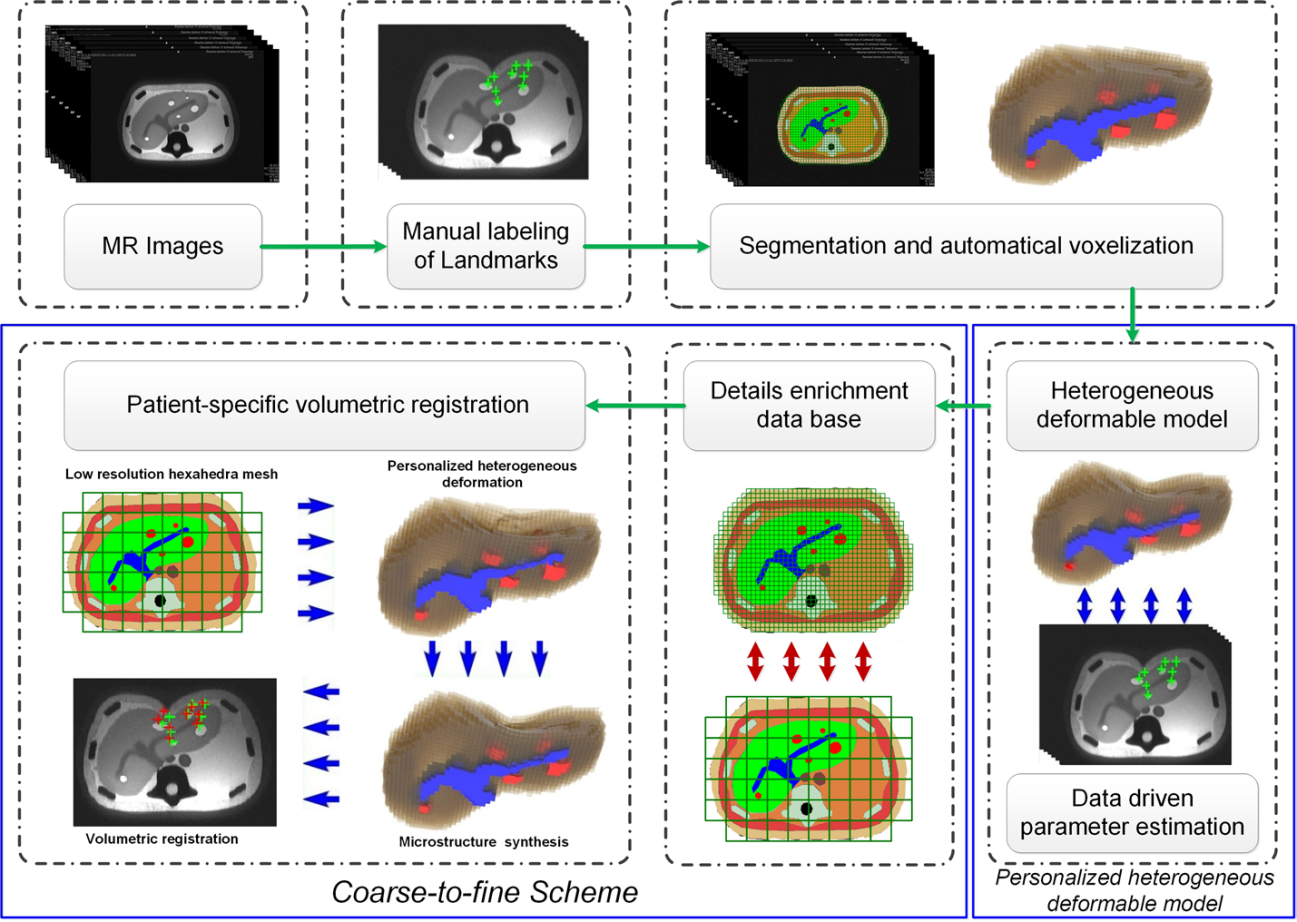
Complete pipeline of personalized heterogeneous deformable model for real-time volumetric registration

In this work, we propose a personalized heterogeneous deformable model to accurately and efficiently model the mechanical behavior of human organ under interaction, aiming at achieving fast volumetric registration. The overall process is illustrated in Fig. 3.

#### High-resolution heterogeneous deformable model

Taking liver tissue containing a tumor as an example, the hexahedron containing both part of tumor and soft tissue is called boundary hexahedron. Here the shared vertices of liver hexahedral and tumor hexahedral meshes forms the internal surfaces and these vertices are called internal surface vertices. By representing the internal surface vertices using their neighboring hexahedral vertices, the coupling forces can be transmitted to the neighboring hexahedra vertices whose displacements can also be reflected on the internal surface vertices.

**Fig. 4:**
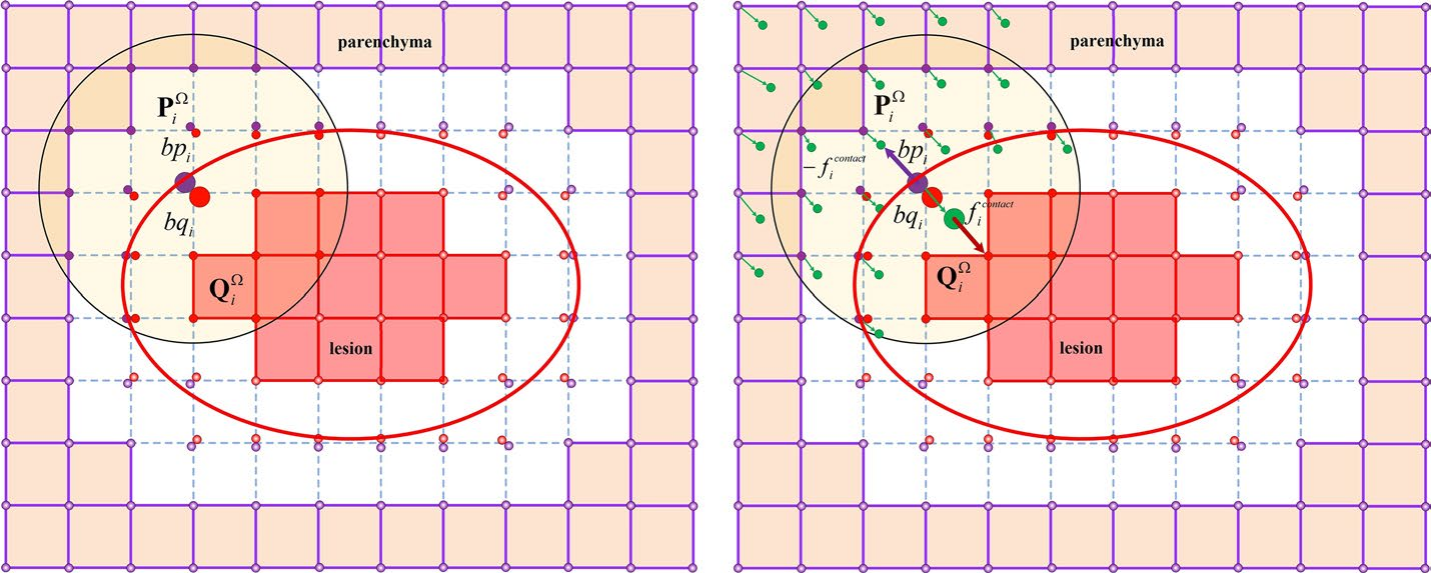
Heterogeneous structure of deformable model. The *purple line* is the parenchyma and the *red line* is the lesion. The boundary vertices are splitted into two vertices which respectively represent parenchyma and lesion. The *imaginary line* represents that there are now springs and the boundary vertices not involved in the TLED FEM modeling, which are represented by its neighboring vertices using MLS method. The *green points* represent the new positions of vertices on the hexahedral model in the next time step

As shown in Fig. 4, purple vertices are parenchyma (such as liver soft tissue), red vertices are lesion (such as tumors), which are separated by the internal surface. Here we define $\mathbf {P}=\{p_1,p_2,...\}$ as the parenchyma vertices, $\mathbf {Q}=\{q_1,q_2,...\}$ as the lesion vertices. As the parenchyma and the lesion are coupled all the time on the boundary, here we treat boundary vertex *i* as two corresponding splitted vertices $bp_i$ and $bq_i$, called *boundary vertices*, which are on the same position and obviously they should keep in contact at every time step. We define $\mathbf {BP}=\{bp_1,bp_2,...\}$ as boundary vertices of parenchyma and $\mathbf {BQ}=\{bq_1,bq_2,...\}$ as boundary vertices of lesion. The high-resolution heterogeneous deformation is described as follows: first, we exert a displacement by the interactive tool on the parenchyma vertices and the new position of each parenchyma vertex is solved with TLED FEM. The boundary vertices are not involved in the TLED FEM modeling and they are represented by its support domain using moving least-squares (MLS) [30], as shown in Fig. 4.

Suppose two corresponding boundary vertices $bp_i$ and $bq_i$, $\mathbf {P}_i^\Omega$ and $\mathbf {Q}_i^\Omega$ are their support domain, respectively. At time step *t*, after applying boundary condition on the parenchyma vertices, the parenchyma boundary vertices and lesion boundary vertices are calculated by the following equation.

1 $$\begin{aligned} bp_i^t=\Phi _{p,i}\mathbf {P}_i^{\Omega ,t},\quad bq_i^t=\Phi _{q,i}\mathbf {Q}_i^{\Omega ,t} \end{aligned}$$

where $\Phi _{p,i}, \Phi _{q,i}$ are shape functions according to the MLS method.

During the mechanical coupling procedure, $bp_i$ and $bq_i$ suffer a coupling force and induce lesion to deform. Here we propose a tissue–tissue coupling strategy based on penalty-based method [31], which is a computationally simple and effective solution for collision response between parenchyma, tumor and vessels. The coupling force is

2 $$\begin{aligned} f_{i,t}^{coupling}=-k_{c}\delta ^t \end{aligned}$$

where $\delta ^t=bp_i^t-bq_i^t$ is the interpenetration of parenchyma and lesion, $k_{c}$ is a personalized tissue–tissue coupling coefficient to calculate coupling force in heterogeneous deformable model. For computing the coupling forces between parenchyma and tumor, parenchyma and vessels, $k_{c}$ is represented by $k_{c,s-t}$ and $k_{c,s-v}$, respectively.

After obtaining the coupling force between lesion boundary vertex $bq_i$ and parenchyma boundary vertex $bp_i$, we transmit the coupling force to their support domain $\mathbf {Q}_i^{\Omega ,t}$ and $\mathbf {P}_i^{\Omega ,t}$, respectively.

3 $$\begin{aligned} \mathbf {F}_{q,i}^t=f_{i,t}^{coupling}\Phi _{q,i},\quad \mathbf {F}_{p,i}^t=-f_{i,t}^{coupling}\Phi _{p,i} \end{aligned}$$

Then the parenchyma vertices and lesion vertices are solved with the TLED FEM and the support domains of boundary vertices $bp_i$ and $bq_i$’s are updated as $\mathbf {P}_i^{\Omega ,t+1},$ $\mathbf {Q}_i^{\Omega ,t+1},$ respectively. The positions of $bp_i$ and $bq_i$ in the time step $t+1$ are

4 $$\begin{aligned} bp_i^{t+1}=\Phi _{p,i}\mathbf {P}_i^{\Omega ,t+1},\quad bq_i^{t+1}=\Phi _{q,i}\mathbf {Q}_i^{\Omega ,t+1} \end{aligned}$$

**Fig. 5:**
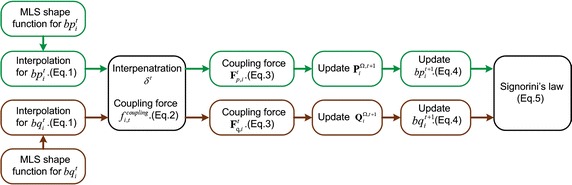
The workflow of the high-resolution heterogenous deformable model

Actually, on the boundary, the parenchyma and lesion are coupled all the time, and they satisfy the Signorini’s law [32]:

5 $$\begin{aligned} 0\le \delta ^{t+1} \perp f_{i,t+1}^{coupling}\ge 0 \end{aligned}$$

where $\perp$ indicates there is a complementarity relation between $\delta ^{t+1}$ and $f_{i,t+1}^{coupling}.$ The workflow of the high-resolution heterogenous deformable model is illustrated in Fig. 5.

#### Data driven parameters estimation

To precisely model volumetric deformation, we have to obtain the tissue–tissue coupling parameters for our heterogeneous deformable model. It is unrealistic to find universal coefficients $k_{c,s-t}$ and $k_{c,s-v}$ which fit all the patients and circumstances [33]. There is an important variation of values when it comes to the parameters estimation of our deformable model. The parameters should be selected according to the personalized application.

From the experimental data, we adopt 3 datasets of compression for parameters estimation and the other 2 datasets of compression for validation through calculating the landmarks’ registration errors. For each dataset for parameter estimation, deformation can be described as:

6 $$\begin{aligned} \mathbf {X}^{t+1}=\mathbf {K}(k_{c,s-t},k_{c,s-v})\mathbf {X}^t \end{aligned}$$

where $\mathbf {X}^{t+1}$ is the deformed position set of vertices on high-resolution heterogeneous deformable model. $\mathbf {X}^t$ are the vertices on undeformed high-resolution heterogeneous deformable model. $\mathbf {K}(k_{c,s-t},k_{c,s-v})$ are the tissue–tissue coupling parameters of the high-resolution heterogeneous deformable model, and they are obtained by the following equation,

7 $$\begin{aligned} \mathop {\arg \min }\limits _{k_i\in \mathbf {K}} \{\Vert \mathbf {X}_{g}^{t+1}- \mathbf {K}(k_{c,s-t},k_{c,s-v})\mathbf {X}^t\Vert _2\} \end{aligned}$$

where $\mathbf {X}_{g}^{t+1}$ is the groundtruth set of 25 landmarks. The parameters are trained by pairs of the landmarks positions on the high-resolution heterogeneous deformable model.

#### Dynamics

In this paper, we model the dynamics of human organ using the following mathematical formulation:

8 $$\begin{aligned} \mathbf {M}{\ddot{\mathbf {X}}}+\mathbf {D}\dot{\mathbf {X}}+\mathbf {K}(\mathbf {X})\mathbf {X}=\mathbf {R} \end{aligned}$$

where $\mathbf {M}$ is the constant mass, $\mathbf {X}$ is the current nodes’ displacements. $\mathbf {D}(\dot{\mathbf {X}})$ is the damping force, $\mathbf {K}(\mathbf {X})\mathbf {X}$ represents elastic force, and $\mathbf {R}$ is the current applied force field equivalently distributed to each node of the object’s mesh. In addition, we model the deformable body as Neo-Hookean material.

### Coarse-to-fine scheme

**Fig. 6:**
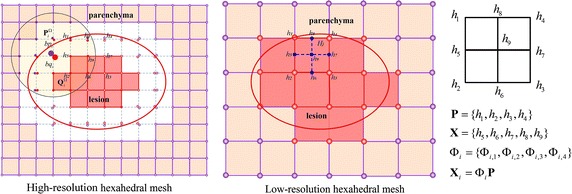
High-resolution hexahedral mesh and low-resolution hexahedral mesh

The high-resolution heterogeneous deformable model can provide a promising way for volumetric registration with high fidelity. However, it needs too much computational cost. To achieve fast volumetric registration, we propose a coarse-to-fine scheme which numerically coarsens the high-resolution hexahedral mesh into a low-resolution hexahedral mesh of $24\times 18\times 18$. Each vertex on low-resolution hexahedral mesh can find a corresponding vertex on the high-resolution hexahedral mesh. As shown in Fig. 6, in a 2D illustration, $h_1, h_2, ... , h_9$ are vertices of the high-resolution hexahedral mesh. After constructing the low-resolution hexahedral mesh, $\mathbf {P}=\{h_1, h_2, h_3, h_4\}$ are served as the vertices of low-resolution hexahedron $H_j$, $\mathbf {X}=\{h_5, h_6, h_7, h_8, h_9\}$ are the microstructures which are synthesized by MLS method, $\Phi _i$ are shape functions.

However, there is a difference between high-resolution and low-resolution hexahedral meshes. For the high-resolution hexahedral mesh, the parenchyma and lesion are separated from each other by the internal boundary as shown as the two splitted vertices and imaginary line, which have no direct connection with the parenchyma or lesion hexahedral mesh. The force transmission between parenchyma and lesion is realized by the internal boundary vertices using MLS method. For the low-resolution hexahedral mesh, the parenchyma hexahedral and lesion hexahedral meshes are connected directly and force transmission between them is also direct. For each material’s hexahedral mesh, we assign different Young’s modulus for them, each hexahedron’s tissue type is determined by the largest number of pixel inside that hexahedron. To achieve accurate volumetric registration with low-resolution hexahedral mesh, we store the mapping relationship between vertices on high-resolution hexahedral mesh and corresponding low-resolution hexahedral mesh using MLS method on different compression conditions (respectively 50 different displacements and 20 different orientations at each of the 5 compression positions) to build a detail enrichment database.

At runtime, we can employ the low-resolution hexahedral mesh to achieve fast first-step deformation, and then synthesize the microstructures according to the detail enrichment database. During the construction of detail enrichment database, each hexahedron on low-resolution hexahedral mesh is regarded as an element. Besides, we can use the 8-vertices on low-resolution hexahedral mesh to describe all the vertices in corresponding high-resolution hexahedral mesh by MLS method. For the *t*-th displacement, the *i*th 8-vertices element $H_i$’s all shape functions can be calculated using MLS method, donated as,

9 $$\begin{aligned} \Phi _{j,t}=(\phi _{j,1},\Phi _{j,2},...,\Phi _{j,8})^{(t)},\quad j\in H_i \end{aligned}$$

After constructing the detail enrichment database, for a specific displacement exerted on the phantom we need to find out the corresponding deformation information under the similar compression conditions in the detail enrichment database to synthesize the microstructures. Suppose the *i*th 8-vertices element $H_i$’s 8 vertices are $\mathbf {P}_i=(P_{i,1},P_{i,2},...,P_{i,8})$, for low-resolution hexahedral mesh element $H_c$, the microstructures can be calculated as,

10 $$\begin{aligned} x_j=\Phi _{j,t}^T \mathbf {P}_i \end{aligned}$$

**Fig. 7:**
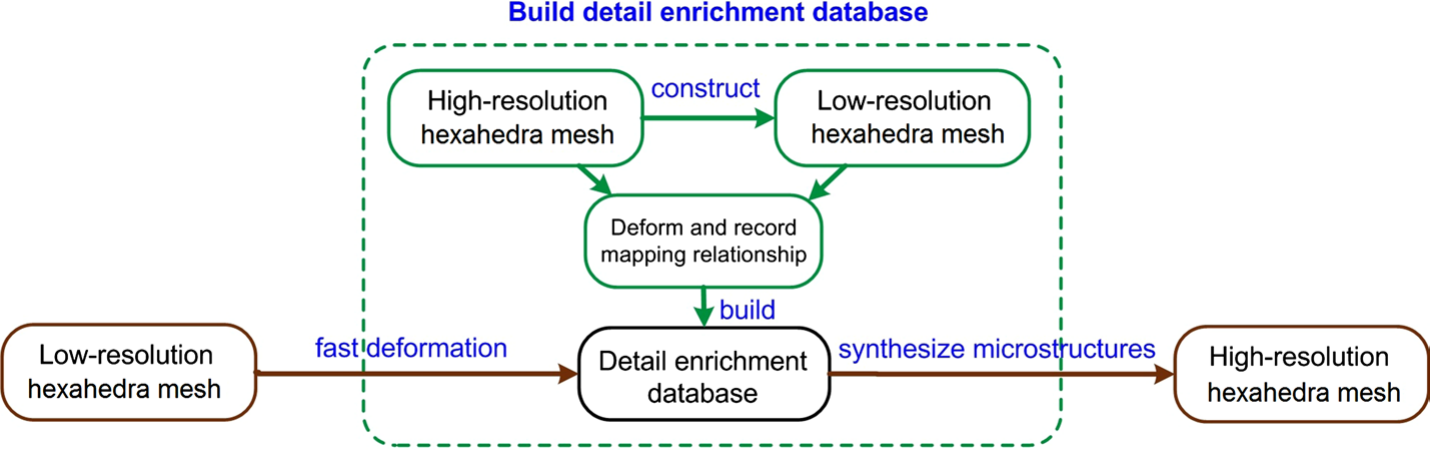
The mechanism of the coarse-to-fine scheme

Figure 7 explains the mechanism of how to construct the mapping between two levels of hexahedral meshes. First, we construct the low-resolution hexahedral mesh according to the high-resolution hexahedral mesh and calculate the mapping relationship by MLS method [30]. Then, we build the detail enrichment database by deforming the high-resolution heterogeneous model and recording the mapping relationship between high-resolution and low-resolution hexahedral meshes. Finally, we compute the deformation of the low-resolution hexahedral mesh, and synthesize the microstructures to achieve the final deformation of the high-resolution hexahedral mesh.

## Experiments

We conducts several experiments to validate our method mainly from two perspectives: accuracy and efficiency. Young’s modulus for parenchyma, vessel and tumor used in the phantom are respectively $2\times 10^5$, $10^6$ pa and $5\times 10^6 pa$ pa, and the Poisson’s ratio for them are 0.49. All experiments are conducted on a PC equipped with Intel Xeon CPU E3-1230 V2 (3.30GHz) CPU, 4G RAM and NVIDIA GeForce GTX 650 Ti.

**Fig. 8:**
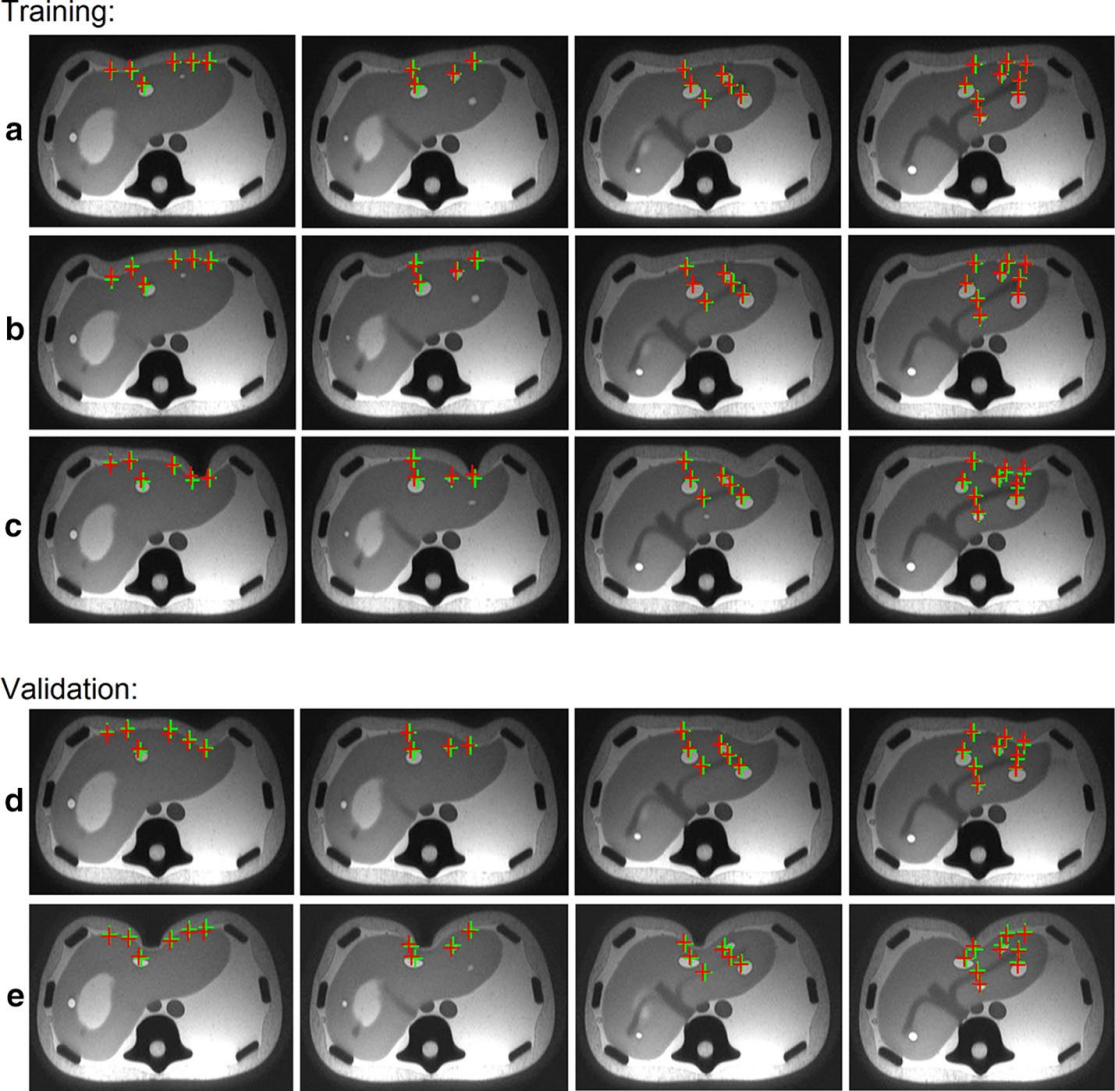
Parameters estimation and validation of personalized heterogeneous deformable model for volumetric liver registration. The *red crosses* represent the calculated results by our method, and the *green crosses* are the ground truth. Here rows **a**, **b**, **c**, **d** and **e** are the selected images of the 5 scanned MRI datasets, which are obtained from the 5 compression experiments on the phantom. The resolution of this high-resolution deformable model is $74\times 18\times 54$

**Data-driven parameters estimation** We divide the five compressions datasets into two groups (three training datasets and two validation datasets). Figure 8 illustrates the volumetric liver registration results on training datasets (*A*, *B* and *C*) and validation datasets (*D* and *E*). Each row represents the data from one compression position. For the phantom, we have labeled 25 landmarks on five cross sections, which are corresponding to the five column in Fig. 8. Each column represents a MR image section with landmarks. To estimate the personalized parameters accurately, we iteratively conduct the deformation experiments for high-resolution heterogeneous deformable model until obtaining the minimum average target registration error (TRE) for training datasets. It is observed from the training datasets *A*, *B* and *C* in Fig. 8 that the calculated results (red crosses) are consistent with the groundtruth (green crosses). The minimum average TRE of the training datasets is 2.00 mm. The estimated tissue–tissue coupling parameters $k_{c,s-t}$ and $k_{c,s-v}$ are 600 and 500 N/m respectively. The average computation time per frame for high-resolution heterogeneous deformable model is 344.83 ms and the average frame rate is 2.9 fps. By applying the estimated parameters on validation datasets, we obtain the volumetric registration results with average TRE 2.55 mm, which demonstrates the effectiveness of the estimated personalized parameters.

**Fig. 9:**
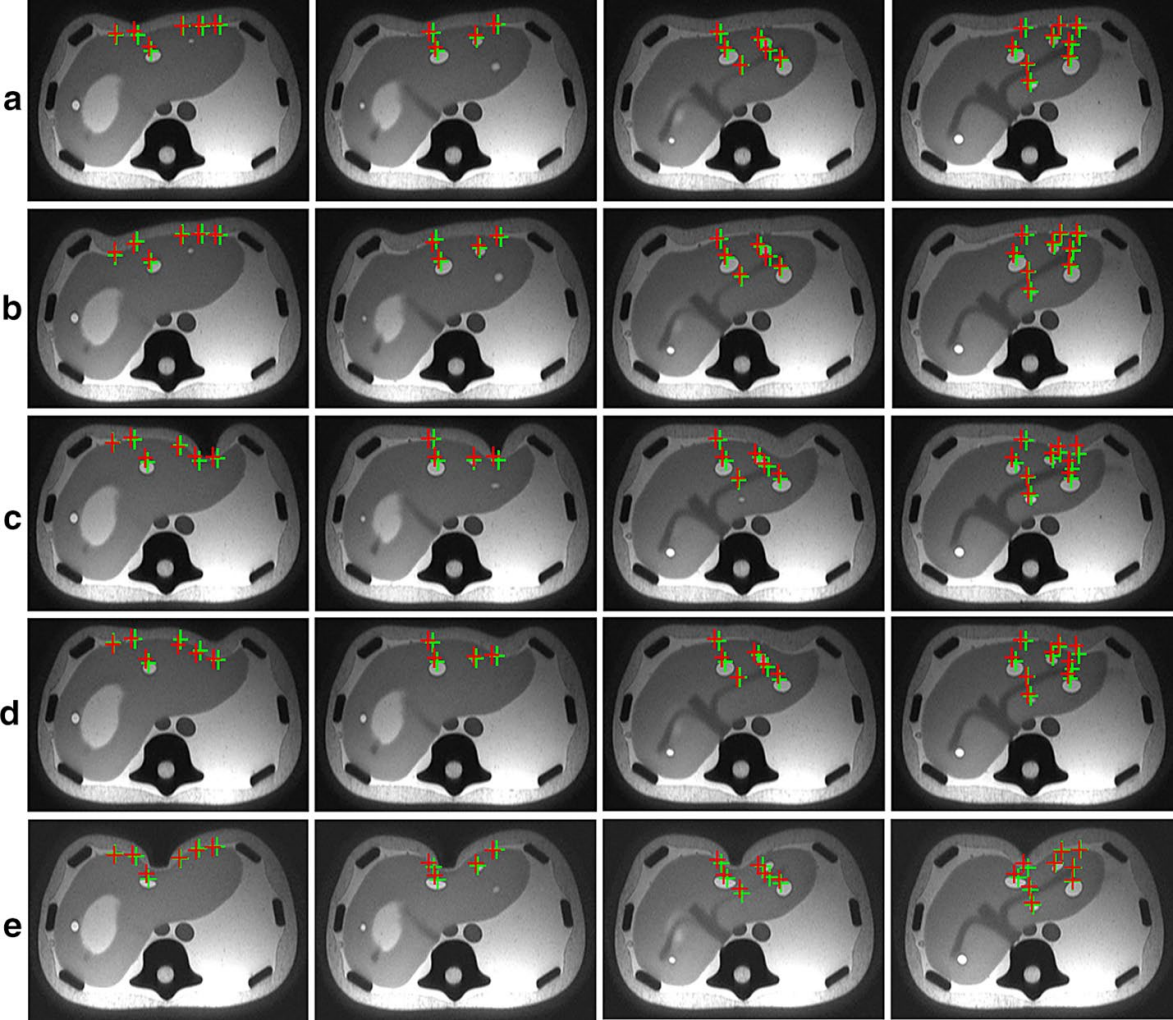
Volumetric liver registration results by our method. The *red crosses* represent the calculated results by our method, and the *green crosses* are the ground truth. Here rows **a**, **b**, **c**, **d** and **e** are the selected images of the 5 scanned MRI datasets, which are obtained from the 5 compression experiments on the phantom. The resolution of low-resolution hexahedral model is $24\times 18\times 18$

**Fig. 10:**
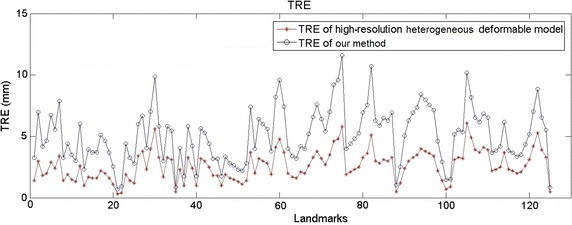
TRE comparison of high-resolution heterogeneous deformable model and our method

**Fig. 11:**
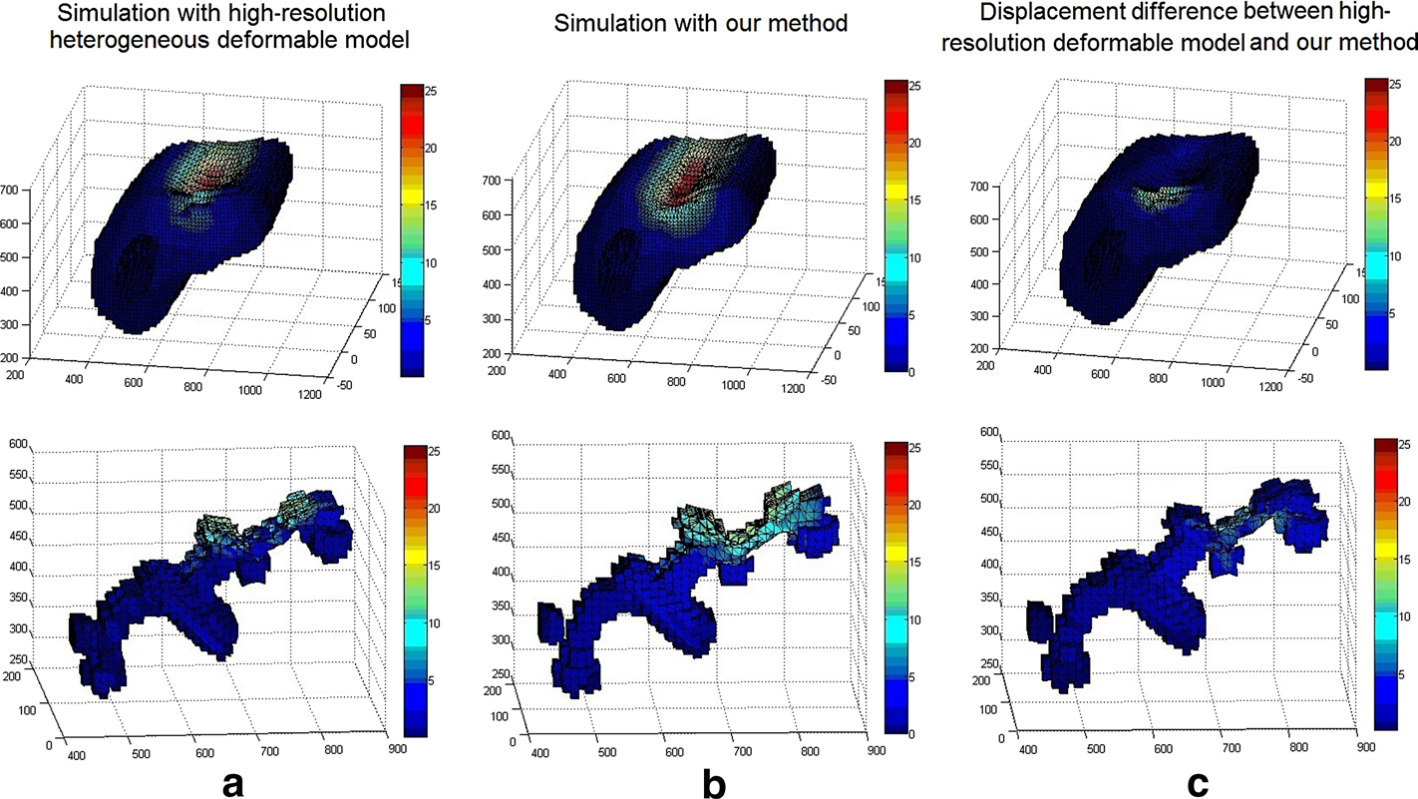
Deformation results comparison using high-resolution heterogeneous deformable model and our method. The *color bar* in **a** and **b** represent the vertices displacements by high resolution deformable model and our method respectively, while the *color bar* in **c** represents the difference of vertices displacements between high resolution deformable model and our method

**Coarse-to-fine scheme** We build a low-resolution deformable model and construct a details enrichment dataset by deforming high-resolution heterogeneous deformable model and recording the mapping relationship using the MLS method. In our method, we direct perform deformation on low-resolution deformable model and synthesize the microstructures according to the detail enrichment dataset. The deformation results of our method (low-resolution deformable model with detail enrichment database) are illustrated in Fig. 9, which demonstrates that the calculated results are consistent with the groundtruth. Figure 10 shows the TRE of high-resolution heterogeneous deformable model and our method, respectively. We demonstrate the deformation comparisons in volumetric registration for high-resolution heterogeneous deformable model and our method. At equilibrium we obtained the displacements of both solutions for dataset *E* (see Fig. 11a, b). Then we measured the difference of the displacements computed by the two approaches. Results are presented in Fig. 11c (using the same scale as the initial displacements). The average TRE is 3.27 mm, which indicates the coarse-to-fine strategy can achieve registration results with good accuracy.

**Fig. 12:**
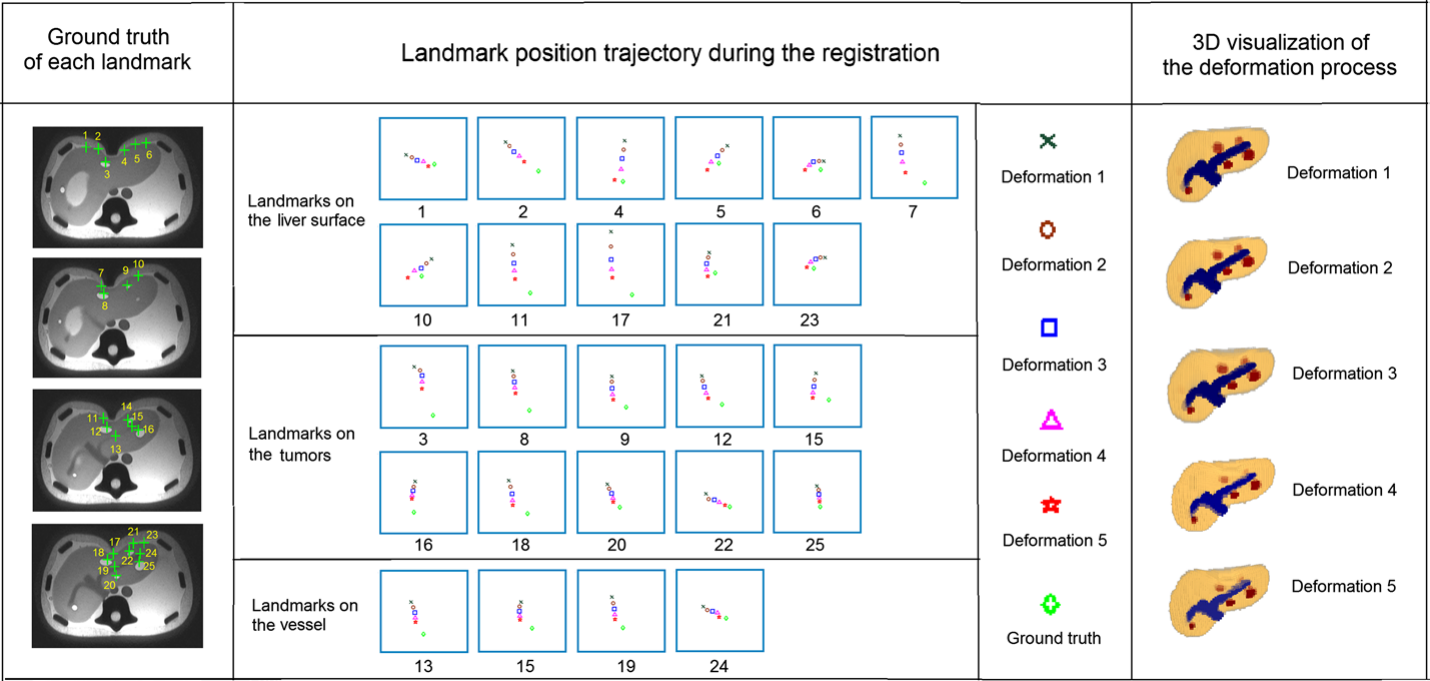
3D visualization of the phantom’s deformation and the landmarks’ deformation trajectory

In addition, we visualize the intermediate deformation poses of our hexahedral mesh and the corresponding positions of the 25 landmarks in this progress. As shown in Fig. 12, we divided the 25 landmarks into three types: surface landmarks, tumor landmarks and vessel landmarks, since these three kinds of landmarks are doctor’s structures of interest. We compare the positions of the 25 landmarks in these deformed model with the ground truth, which indicates that the calculated landmark positions are approaching the ground truth gradually during the registration process.

**Comparison** We validate our method (low-resolution deformable model with detail enrichment database) from the following perspectives:

- Compare the overall registration accuracy and performance of our method with the registrations using high-resolution heterogeneous deformable model and Han et al. [13], which is also a non-rigid registration method based on the TLED FEM while without considering the tissue heterogeneity.
- Compare the surface and internal registration results of our method with the above two registration methods respectively, demonstrating the accuracy of heterogeneity representation of our method.

**Fig. 13:**
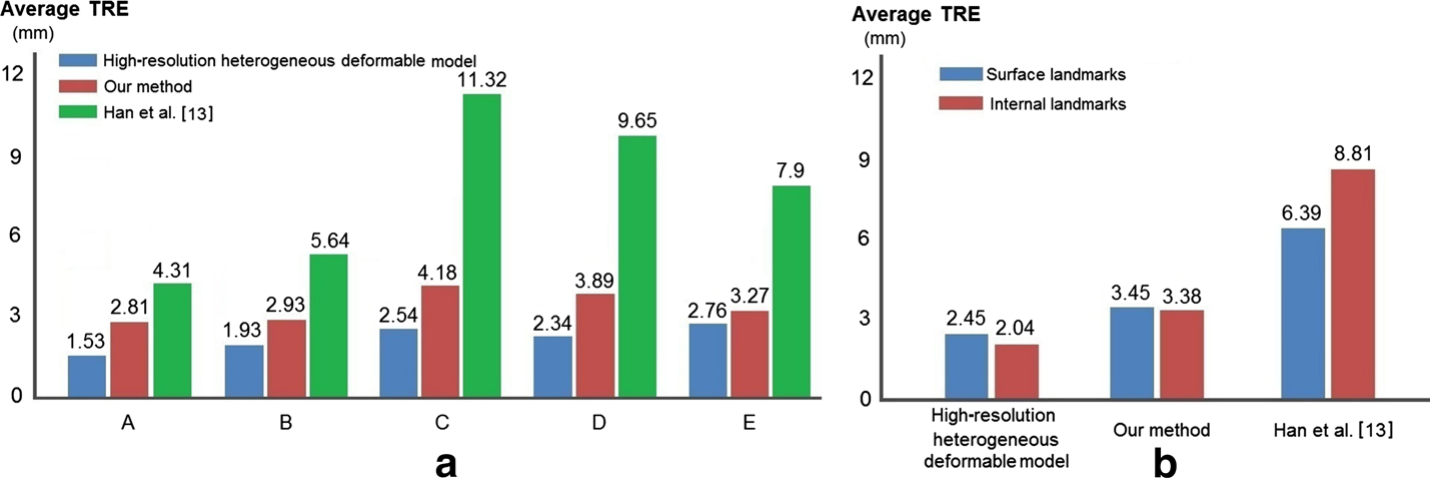
Average TRE of high-resolution heterogeneous deformable model, our method and Han et al. [13] respectively

Figure 13a illustrates the average TRE of volumetric registration on our method, high-resolution heterogeneous deformable model and Han’s work [13] for datasets *A*, *B*, *C*, *D* and *E*. It can be observed that the average TRE of Han’s method (7.74 mm) for datasets *A*, *B*, *C*, *D* and *E* are all larger than those of high-resolution heterogeneous deformable model (2.22 mm) and our method (3.42 mm). Because the high-resolution heterogeneous deformable model includes the tissue–tissue coupling process in the volumetric mesh, which results in less registration error than Han’s method, which did not include the tissue–tissue coupling process. Besides, our method first deforms the low-resolution hexahedral mesh and then synthesizes the microstructures of the high-resolution hexahedral mesh according to the detail enrichment database, which is constructed by the mapping relationship of the deformed high-resolution heterogeneous deformable model and low-resolution deformable model. The detail enrichment database itself already includes the effects of the tissue–tissue coupling process.

Meanwhile, we evaluate the registration accuracy of 11 surface landmarks and 14 internal landmarks on the phantom model respectively, as shown in Fig. 13b. For high-resolution heterogeneous deformable model, the average TRE of surface landmarks and internal landmarks are respectively 2.45 and 2.04 mm. While for our method, the average TRE of surface landmarks and internal landmarks are 3.46 and 3.38 mm, respectively. Using Han’s method, the average TRE of surface landmarks and internal landmarks are 6.39 and 8.81 mm, respectively. Experimental results indicate our method can well describe the heterogeneity of human organ, and achieve better registration accuracy than Han’s method [13].

In addition, we achieve 27.2 fps of volumetric registration by our method with the average computation time 36.76 ms, this frame rate can fulfill the requirement of real-time tracking system. It is also worth noting that at the expense of accuracy for about 1 mm, our method can speed up $9.4\times$ than the high-resolution heterogeneous deformable model, as well as about $9.4\times$ faster than Han’s method which is applied on the high-resolution deformable model. The experimental results demonstrate that our method can well balance the computational efficiency and accuracy.

## Discussion and conclusion

In this paper, an efficient personalized heterogeneous deformable model is presented for volumetric registration. Our method includes three core components: a heterogeneous deformable model, a personalized tissue–tissue coupling strategy and a coarse-to-fine scheme. In more detail, we propose the high-resolution heterogeneous deformable model for a uniform and high-resolution hexahedral mesh and model the mechanical behavior of heterogeneous anatomical structure with TLED FEM and penalty method. Besides, we present a data driven parameters estimation method for the high-resolution heterogeneous deformable model to obtain the tissue–tissue coupling parameters of our method in vivo for FE analysis. In addition, we put forward a coarse-to-fine scheme to achieve fast volumetric registration, which is to first perform the volumetric deformation on the low-resolution hexahedral mesh and then synthesize the microstructures according to the detail enrichment database. We have tested the high-resolution heterogeneous deformable model and our method with the real compression of phantom in five experiments. The experimental results indicate our method can achieve fast and accurate registration results, which are essential for clinical applications.

Biomechanical deformable model is an effective way to reliably predict deformation for volumetric registration and many researchers have demonstrated good non-rigid registration that meets the accuracy requirements of specific surgery. Al-Mayah et al. [17] proposed a 3D FEM based biomechanical model for image registration of head and neck cancer treatment, they applied the linear elastic material properties to their method and adopted linear geometry. Oktay et al. [16] proposed a linear FEM deformation based registration method for pre-operative and intra-operative 3D image fusion for laparoscopy surgery. Though the linear FE analysis is an approximation that makes the analysis of the structure more tractable, the assumptions of linearity are often not adequate for real tissues which often undergoes nonlinear behaviour. Compared with the work of  [16, 17], our TLED FEM analysis adopts nonlinear elastic material properties which can provide more accurate biomechanical analysis. Hopp et al. [15] presented a nonlinear biomechanical FEM analysis based registration method for X-ray mammograms with DCE-MRI volumes. The mean TRE was 13.2 mm that was within the clinically relevant range. However, the deformable body was modelled as homogeneous soft tissue which could not describe the deformation distribution inside the soft tissue. Thus their method is not suitable for the registration of organs with internal heterogeneous structures such as tumors or vessels. To address the heterogeneous issue, Haouchine et al. [11] used a deformable volumetric biomechanical model accounting for heterogeneity and anisotropy in hepatic surgery guidance with the best tumor registration accuracy of 2.5 mm. Samavati et al. [18] proposed a biomechanical model with heterogenous material property for deformable prostate image registration with average registration accuracy of 4.8 mm, also they have presented a hybrid biomechanical intensity based deformable image registration method for lung 4DCT [19] and achieved average registration accuracy of 2.9 mm. Han et al. [13] proposed a patient-specific biomechanical modeling framework for heterogeneous breasts based on nonlinear FEM solver, which achieved relative accurate volumetric breasts registration with the best registration accuracy of $3.18\pm 1.69$ mm by anisotropic heterogeneous model. They assigned different material properties for different tissues to construct the heterogeneous structures. Different from the above heterogenous models, our method constructs the heterogeneous deformable on a uniform and high-resolution hexahedral mesh directly extracted from the MR images for investigating the motion of liver’s vessels and tumors, and the different types of tissues are coupled by a penalty method. The proposed high-resolution heterogeneous deformable model achieves average registration accuracy of 2.22 mm on a fine resolution grid of $74\times 18\times 54$. Our method achieves average registration accuracy of 3.42 mm on a low-resolution grid of $24\times 18\times 18$ with detail enrichment database, while the method of Han et al. [13] achieves TRE of 7.74 mm.

In spite of high registration accuracy achieved by our heterogeneous model, the efficiency due to the tremendous computation has limited the applications of many work [11, 13, 18, 19], whose registration process is time-consuming. However, the efficiency is an essential issue in the image-guided surgery for the reason that even in a few seconds the registration target would deform or shift a lot which could lead to the failure of the registration. It is crucial to develop a fast biomechanical model based registration methods which incorporate the advantages of high accuracy and efficient computation. To achieve fast volumetric registration, we propose a practical coarse-to-fine scheme and establish a detail enrichment database at the preprocessing stage. At runtime, we simulate the mechanical behavior of the low-resolution hexahedral mesh and synthesize the micro-structures in virtue of detail enrichment database. With the expense of accuracy for about 1mm, our method can speed up $9.4\times$ than the high-resolution heterogeneous deformable model.

There are certain limitations of our method. In clinical practice, the personalized Young’s modulus is unknown while it is very essential for the construction of personalized deformation model. We plan to obtain the patient-specific Young’s modulus by ultrasound elastography in vivo [34]. Besides, the accuracy of the low-resolution deformable model is not as accurate as the high-resolution one, so that we plan to incorporate the generalized moving least squares (GMLS) [35] into our coarse-to-fine scheme to improve its accuracy. In addition, we intend to improve the setup of boundary conditions for the temporal registration phase, which is an important and challenging problem. Besides, we will also adopt our method to align the pre-operative volumetric liver MR images to intra-operative ultrasound image and obtain quantitative errors on real data.

## Abbreviations

CT: computed tomography
MR: magnetic resonance
MRI: magnetic resonance imaging
FEM: finite element method
TLED: total Lagrangian explicit dynamics
MLS: moving least squares
GMLS: generalized moving least squares
TRE: target registration error
DCE-MRI: dynamic contrast-enhanced magnetic resonance imaging
4DCT: 4-dimension computed tomography
